# Olanzapine Overdose in an Adult Male Presenting With Altered Sensorium and Pinpoint Pupil: A Case Report

**DOI:** 10.7759/cureus.100538

**Published:** 2025-12-31

**Authors:** Mohammad Abdurrahman Khan, Manisha Verma, Kahkashan Riaz

**Affiliations:** 1 Department of Forensic Medicine and Toxicology, Hind Institute of Medical Sciences, Barabanki, Lucknow, IND; 2 Department of Dentistry, Hind Institute of Medical Sciences, Barabanki, Lucknow, IND; 3 Department of Pathology, Integral Institute of Medical Sciences and Research, Lucknow, IND

**Keywords:** antipsychotic, olanzapine, overdose, poisoning, toxicity

## Abstract

Olanzapine is an atypical antipsychotic prescribed for various mental illnesses such as psychosis, schizophrenia, depression, and bipolar disorder. An overdose of olanzapine leads to many adverse effects, such as agitation, tachycardia, extrapyramidal dystonia, dysarthria, blurred vision, myosis, hypotension, respiratory depression, and deep coma. Yet, no antidote is available for olanzapine poisoning, and, hence, supportive care is a mainstay of management. A 51-year-old male presented to the emergency department in an unconscious state with an alleged history of drug overuse, with the ingestion of 10 tablets of olanzapine 10 mg. The patient was intubated. After intubation, Ryle’s tube was inserted, and gastric lavage was performed using 300 mL of normal saline. The rest of the treatment was supportive. Prompt clinical evaluation, along with thorough history-taking from patients or family members, is a crucial step in patient management and thereby improving the outcome. Increasing awareness among healthcare providers about toxicity due to olanzapine overdose is an important step for assuring early intervention and improving patient care.

## Introduction

Olanzapine is an atypical antipsychotic drug that belongs to the thienobenzodiazepine group. It is widely prescribed for various mental illnesses such as psychosis, schizophrenia, depression, and bipolar disorder, along with several off-label clinical conditions such as anorexia nervosa, delirium, agitation, and for controlling chemotherapy-induced nausea and vomiting in children [1,2]. Olanzapine has antipsychotic activity because it antagonizes dopamine receptors, reducing dopamine-mediated symptoms. The range of clinical signs during its overdose or acute poisoning is due to its actions at multiple receptors, including antagonism at dopamine (D1, D2, D4-reducing dopamine activity), histamine H1 (causing sedation), serotonin 5-HT2A and 5-HT2C (affecting mood and cognition), muscarinic cholinergic M1-M5 (producing anticholinergic effects), and adrenergic α1 (leading to hypotension) receptors [3]. Olanzapine overdose leads to many adverse effects, such as agitation, tachycardia, extrapyramidal dystonia, dysarthria, blurred vision, myosis, hypotension, respiratory depression, and deep coma [2]. Its overdose also alters laboratory parameters such as leukocytosis, hyperglycemia, electrolyte imbalance, and conduction abnormalities on ECG [1]. Yet, no antidote is available for olanzapine poisoning, making supportive care the mainstay of the management. Severity of poisoning correlates with the ingested amount of olanzapine as well as whether olanzapine is co-ingested with additional drugs. [3]. Here, we report a case of olanzapine overdose in an adult male with ingestion of 10 tablets, each with 10 mg of olanzapine.

## Case presentation

A 51-year-old male presented to the emergency department of our tertiary care hospital in an unconscious state with an alleged history of drug overuse, with the ingestion of 10 tablets of olanzapine 10 mg eight hours ago at 10.24 AM. The patient was a known case of generalized anxiety disorder for which he was taking olanzapine 10 mg HS and lorazepam 1 mg HS for three to four years. The patient had no history of diabetes, hypertension, heart disease, chronic obstructive pulmonary disease, kidney disease, tuberculosis, etc. The patient also did not have a similar family history. He had no history of smoking or alcohol consumption.

At the time of admission, his temperature was 36.83°C, pulse was 120 beats/minute, blood pressure (BP) was 100/60 mmHg, respiratory rate (RR) was 28 breaths/minute, and SpO_2_ was 90%. The patient was unconscious, with a Glasgow Coma Scale score of E1V1 M1. His motor and sensory function could not be assessed. All the cranial nerves were intact, pupils were bilaterally constricted, plantars were bilaterally flexor, cardiovascular examination was normal, respiratory examination was normal (bilateral air entry was present, no added sound was present, trachea was centrally placed, and breathing was abdominothoracic), and abdominal examination was normal.

The patient was intubated after obtaining informed consent from a family member. Post-intubation, pulse was 84 beats/minute, BP was 110/70 mmHg, RR was 26 breaths/minute, SpO_2_ was 98% on ventilator, and temperature was 36.11°C. After intubation, Ryle’s tube was inserted and aspirated with a 50 mL syringe, and the fluid was sent for forensic evaluation. Gastric lavage was performed using 300 mL of normal saline. Fluid returning through Ryle’s tube was dark yellow. Besides this, activated charcoal was also used. On days one to three, the patient was managed according to the treatment outlined in Table 1.

**Table 1:** Treatment administered on days one, two, and three.

Day 1	Day 2	Day 3
Injection (amoxicillin + clavulanic acid) 1.2 g IV TDS	Injection (amoxicillin + clavulanic acid) 1.2 g IV TDS	Injection (piperacillin + tazobactam) 4.5 g IV TDS
Injection ceftriaxone 1 gm IV BD	Injection metronidazole 0.5 g IV TDS	Injection metronidazole 0.5 gm IV TDS
Injection pantoprazole 40 mg IV BD	Injection pantoprazole 40 mg IV BD	Injection pantoprazole 40 mg IV BD
Injection ondansetron 4 mg IV BD	Injection ondansetron 4 mg IV BD	Injection ondansetron 4 mg IV BD
Intravenous fluid DNS I unit, and normal saline with multivitamin II unit	Injection paracetamol 100 ml IV SOS	Intravenous fluid DNS I unit with multivitamin, and NS with 40 mEq (2 amp) KCl
Injection NORAD 4 mL + 46 mL NS SOS	Intravenous fluid DNS I unit, and normal saline with multivitamin II unit	Injection paracetamol 100 mL IV SOS

Random blood sugar (RBS) charting was advised every four hours. Ventilator setting is outlined in Table 2.

**Table 2:** Ventilator settings on days one, two, and three. TV = total volume; PEEP = positive end-expiratory pressure

Ventilator setting	Day 1	Day 2	Day 3
TV	420 mL	440 mL	420 mL
FiO_2_	100%	40%	30%
PEEP	6 cm of H_2_O	6 cm of H_2_O	5 cm of H_2_O
Rate	20/minute	14/minute	14/minute

Although a psychiatric reference was sent on the same day, as the patient was on the ventilator, a psychiatric evaluation could not be done.

All hematological parameters are outlined in Table 3. A chest X-ray at this time was normal.

**Table 3:** Hematological parameters at days one to four. TLC = total leukocyte countl; DLC = differential leukocyte count; GBP = general blood picture; SGPT = serum glutamic pyruvic transaminase; SGOT = serum glutamic oxaloacetic transaminase; CRP = C-reactive protein

Hematology	Day 1	Day 2	Day 3	Day 4	Reference value
Hemoglobin	12.9	12.7	-	10.8	12–15 g/dL
TLC	12,000	11,200	-	8,400	4,000–11,000/µL
DLC	71, 27, 02, 00, 00	83, 15, 02, 00, 00	-	93, 06, 01, 00, 00	40–80, 20–40, 1–6, 0–5, 0–2
Platelets count	1.5 lakhs/cc	1.7 lakhs/cc	-	1.5 lakhs/cc	1.5–4.5 lakhs/cc
GBP	Normocytic, normochromic	Normocytic, normochromic	-	-	-
Serum Na^+^	141.1	144.5	145.6	143.5	136–145 mg/dL
Serum K^+^	3.32	3.02	3.98	3.02	3.50–5.10 mg/dL
Serum ionic Ca^++^	0.92	0.92	-	-	1.05–1.30 mmol/L
Serum creatinine	0.73	-	-	-	0.5–1.4
Serum Urea	19	-	-	-	10–45 mg/dL
Direct bilirubin	0.39	-	-	-	0–0.4
Total bilirubin	1.01	-	-	-	0.3–1.4
SGPT	27	-	-	-	0–40 IU/L
SGOT	23	-	-	-	0–40 IU/L
Alkaline phosphatase	88	-	-	-	50–240 IU/L
CRP	-	-	241.5	123.8	<1.0 mg/dL

On day two, BP was 134/86 mmHg, pulse was 106 beats/minute, RR was 20 breaths/minute, SpO_2_ was 99% on ventilator, and temperature was 38.22°C. Stool was not passed at this time. On cardiovascular examination, S1 and S2 were normal, and there was no murmur. The patient was unconscious and disoriented to time, place, and person. His GCS score was E2VTM4. His plantar reflexes were bilaterally flexor, and his pupils were bilaterally sluggish to light. Respiratory examination was normal (bilateral air entry was present, and no added sound was present. His abdominal examination was normal.

On day 3, BP was 126/80 mmHg, pulse was 94 beats/minute, RR was 26 breaths/minute, SpO_2_ was 98% on ventilator, and temperature was 38.61°C. His RBS was 171 mg/dL. On day three, the patient passed stool. On cardiovascular system examination, S1 and S2 were normal, and there was no murmur. The patient was conscious and oriented to time, place, and person. His GCS score was E4VTM6. His pupils were bilaterally reactive to light, and plantar reflexes were bilaterally flexor. Respiratory examination was normal (bilateral air entry was present, with no added sound. His abdominal examination was normal.

The patient’s management on days four to six is outlined in Table 4.

**Table 4:** Treatment given on days four, five, and six.

Day 4	Day 5	Day 6 (at time of discharge)
Injections (piperacillin + Tazobactam) 4.5 g IV TDS	Injections (piperacillin + tazobactam) 4.5 g IV TDS	Tablet cefixime 200 mg BD for 5 days
Injection metronidazole 0.5 gm IV TDS	Injection metronidazole 0.5 g IV TDS	Tablet nitrofurantoin 100 mg BD for 5 days
Injection pantoprazole 40 mg IV BD	Injection pantoprazole 40 mg IV BD	Tablet (risperidone + trihexyphenidyl) 2 mg BD for 7 days
Injection ondansetron 4 mg IV BD	Injection ondansetron 4 mg IV BD	Tablet lorazepam 2 mg HS for 7 days

On day four, BP was 118/70 mmHg, pulse was 70 beats/minute, RR was 24 breaths/minute, SpO_2_ was 96% on room air, and temperature was 36.94°C. His RBS was 171 mg/dL. The patient passed stool. On cardiovascular system examination, S1 and S2 were normal, with no murmur. The patient was conscious and oriented to time, place, and person. His GCS score was E4V5M6. His pupils were bilaterally reactive to light, and plantar reflexes were bilaterally flexor. Respiratory examination was normal (bilateral air entry was present, no added sounds present). His abdominal examination was normal.

On day five. BP was 110/70 mm Hg, pulse was 72 beats/minute, RR was 20 breaths/minute, SpO_2_ was 95% on room air, and temperature was 36.22°C. His RBS was 139 mg/dL. The patient passed stool. On cardiovascular system examination, S1 and S2 were normal, with no murmur. The patient was conscious and oriented to time, place, and person. His GCS score was E4V5M6. His pupils were bilaterally reactive to light, and his plantar reflexes were bilaterally flexor. Respiratory examination was normal (bilateral air entry was present, no added sounds were present). The abdominal examination was normal.

On day six, the patient’s condition was stable, and the patient was discharged on the sixth day. At discharge, the prescribed medications are outlined in Table 4. The patient was asked to review after seven days.

## Discussion

Olanzapine is a commonly used second-generation atypical antipsychotic drug with lower extrapyramidal side effects than first-generation typical antipsychotics [4]. Olanzapine poisoning in clinical settings usually results from psychiatric patients ingesting their prescribed medicine in a suicide attempt. Overdose symptoms vary widely depending on the dose and its multi-receptor action, but are generally characterized by agitation, somnolence, miosis, blurred vision, hypotension, cardiovascular side effects, respiratory depression, deep coma, anticholinergic, and extrapyramidal effects. Pinpoint pupil presents in 30-35% cases of olanzapine overdoses. A similar finding was present in our case as well as in the study reported by Kumar et al. [1]. With coma, mechanical ventilation is required. Our patient, with generalized anxiety disorder, presented unconscious and in respiratory depression, miosis, and coma (GCS 3), necessitating ventilation [5,6]. Cardiovascular side effects typically occur more with first-generation than atypical antipsychotics [4], but olanzapine overdose can still cause dopamine-resistant hypotension, QT prolongation, and fatal arrhythmia in susceptible individuals, though not in our patient [1]. In our case, adequate hydration with normal saline maintained renal function and avoided acute kidney injury, which was similar to that reported by Keval et al. and Bosch et al. [3,7]. While a few studies report dose-dependent muscle toxicity with high-dose ingestion (about 400 mg) and creatine phosphokinase elevation in 17% of cases, our patient’s low dose did not produce this effect [8]. As no specific antidote exists for olanzapine intoxication, management relies on supportive care. Early gastric lavage with activated charcoal helps reduce complications [9]. Our patient received gastric lavage with 300 mL of normal saline along with activated charcoal through a Ryle’s tube after airway stabilization.

## Conclusions

Atypical antipsychotics such as olanzapine are commonly used for psychosis, but there is always a risk of adverse effects due to overdose. In clinical practice, olanzapine poisoning is usually seen in psychiatric patients who ingest their own prescribed medicine in a suicidal attempt. Prompt clinical evaluation, along with history-taking from the patient or family members, is a crucial step in the management and improvement of the outcome. Intubation and ventilatory support leads to quick recovery in olanzapine toxicity, especially in patients with poor GCS. Increasing awareness among healthcare providers about toxicity due to olanzapine overdose is an important step for assuring early intervention and improving patient care. Lack of facility of toxicological confirmation, such as serum olanzapine level, at our tertiary care center is a limitation of this study.
